# Accuracy, Reproducibility, and Gaps in Different Angulations of 3D-Printed versus Milled Hybrid Ceramic Crown

**DOI:** 10.1055/s-0044-1795116

**Published:** 2024-12-30

**Authors:** Nadaprapai Khwanpuang, Chayaporn Suphachartwong, Awiruth Klaisiri, Seelassaya Leelaponglit, Chayanit Angkananuwat, Nantawan Krajangta

**Affiliations:** 1 Division of Restorative Dentistry, Faculty of Dentistry, Thammasat University, Pathum Thani, Thailand; 2 Thammasat University Research Unit in Restorative and Esthetic Dentistry, Faculty of Dentistry, Thammasat University, Pathum Thani, Thailand; 3 Division of Prosthodontic Dentistry, Faculty of Dentistry, Thammasat University, Pathum Thani, Thailand

**Keywords:** CAD/CAM, 3D printed, accuracy, reproducibility

## Abstract

**Objectives:**

This study compared the accuracy, reproducibility, and gap of crowns resulting from variations in print angulation of three-dimensional (3D)-printed VarseoSmile Crown ^plus^ (VS) and milled resin-ceramic hybrid materials (Cerasmart 270, CS, and Enamic, E).

**Materials and Methods:**

A total of 60 specimens, consisting of VS printed at four different angulations (30, 45, 60, and 90 degrees), along with CS and E were investigated. External and internal accuracy and reproducibility were measured with the 3D deviation analysis. External and internal gaps were measured with the silicone replica technique. The results were analyzed using Welch's one-way analysis of variance with Dunnett T3 post hoc comparison at *p* ≤ 0.05.

**Results:**

Across all groups, external and internal accuracy were 0.55 to 20.02 μm and external and internal reproducibility were 0.05 to 0.69 μm. Overall external accuracy was not significant ( *p* = 0.063), whereas significance was noted in overall internal accuracy and reproducibility among groups ( *p* < 0.001). External and internal gaps were 33.76 to 93.11 μm. Statistically significant differences were found in internal and external gaps among groups ( *p* < 0.001), with milled crowns demonstrating larger internal and smaller external gaps than 3D-printed crowns. Within the 3D-printed group, statistically, 90-degree angles exhibited the smallest external and internal gaps.

**Conclusion:**

Both milled and 3D-printed methods achieved clinically acceptable accuracy, reproducibility, and gap dimensions, offering viable options for hybrid ceramic crown restoration. Among 3D-printed crowns, the 90-degree printing angle group exhibited satisfactory accuracy and reproducibility, alongside the best internal and external fit.

## Introduction

Since the introduction of dental computer-aided design/computer-aided manufacturing (CAD/CAM) technology in the 1970s, dentistry has gradually transitioned toward digitalization. CAD/CAM restorations have not only demonstrated clinical success comparable to traditional methods but have also proven to be time-efficient, enhancing patient convenience. 1 The hybrid ceramic crown has gained recognition for its ability to combine the aesthetic and optical characteristics of ceramic with the additional benefit of lowering fragility. This is achieved through the incorporation of resin to attain a modulus of elasticity similar to that of dentine. As a result, it has become an increasingly popular option for indirect restorative materials. 2 Most CAD/CAM restorations are milled, leading to significant material waste. Additive manufacturing (AM), such as Digital Light Processing (DLP), minimizes waste and enables complex shape fabrication. 3 However, early reports indicated lower accuracy compared to milling, and limited material options hindered widespread adoption in dentistry. 4 Nevertheless, recent DLP has gained popularity in dental practice due to its high accuracy, impressive surface texture, and rapid production capabilities. DLP utilizes ultraviolet laser-activated liquid resin and digital micromirror device technology, offering high accuracy, excellent surface texture, and rapid production capabilities by curing entire layers in a single shot. 5

AM creates restorations layer by layer, resulting in anisotropic properties that vary with the direction of the applied force. The printing orientation plays a pivotal role in this phenomenon, directly influencing the overall strength of the printed interim restoration. 6 Furthermore, the orientation of printing angles influences the overall accuracy and reproducibility and also the gap (external and internal) of the interim restoration. 7 8 The implications of print angulation on the accuracy and reproducibility that may affect the external gap (EG) and internal gap (IG) of three-dimensional (3D)-printed hybrid ceramic crowns are not well established. A previous study indicated that a 45-degree angle yields the maximum level of accuracy for specimens with a bar shape. 9 However, the outcome for specimens with a crown shape of hybrid ceramic material remains uncertain. Several previous studies reported inconsistent results for the best angulation of 3D printing to achieve marginal adaptability of 3D-printed specimens. 10 11 12 Therefore, this study aims to compare the external and internal accuracy, reproducibility, and gaps of 3D-printed hybrid ceramic crowns at different angle settings in comparison to milled hybrid ceramic counterparts. The null hypotheses were that external and internal accuracy, reproducibility, and gaps of hybrid ceramic crowns are not different among groups.

## Materials and Methods

**Fig. 1:**
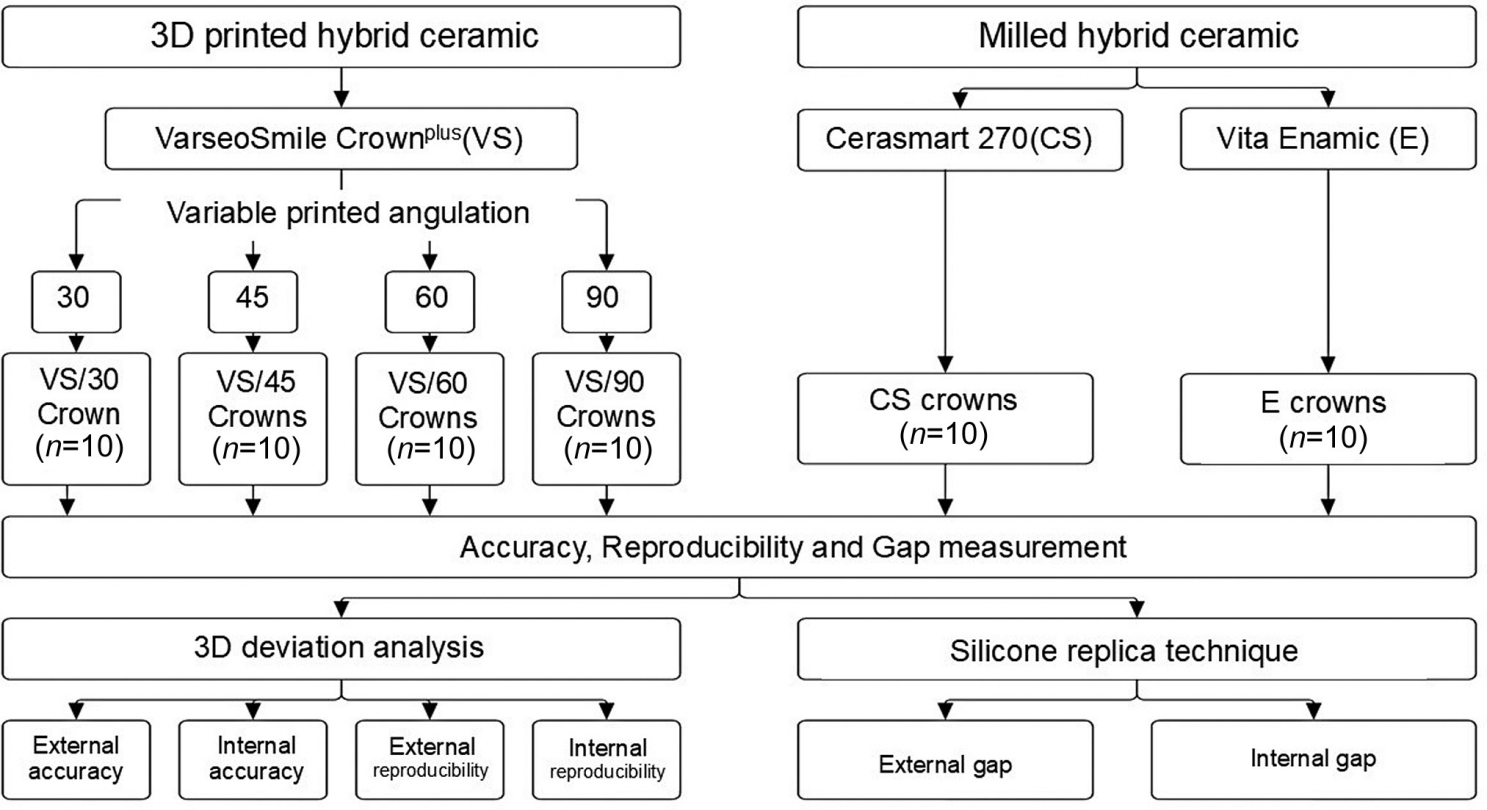
Experimental design.

Specimens were divided into six groups and evaluated for external and internal accuracy, reproducibility, and gaps ( Fig. 1 ). With 10 specimens per group, a power > 0.95 at *α* = 0.05 was achieved, calculated using G*Power 3.1 Software (University of Düsseldorf, Düsseldorf, Germany). The mandibular right molar typodont tooth underwent virtual full crown preparation, incorporating specific dimensions: 1.5 mm occlusal reduction, 1 mm axial reduction, 1 mm heavy chamfer finishing line, and a total occlusal convergence of 12 degrees. The prepared die was 3D scanned using a MEDIT T710 desktop scanner (MEDIT, Seoul, Korea) and processed into a 3D model with Autodesk Fusion 360 (California, United States). Epoxy dies were produced using a Form 3 DLP 3D printer (Formlabs, Massachusetts, United States) with epoxy resin (Rigid 10K, Formlabs).

**Fig. 2:**
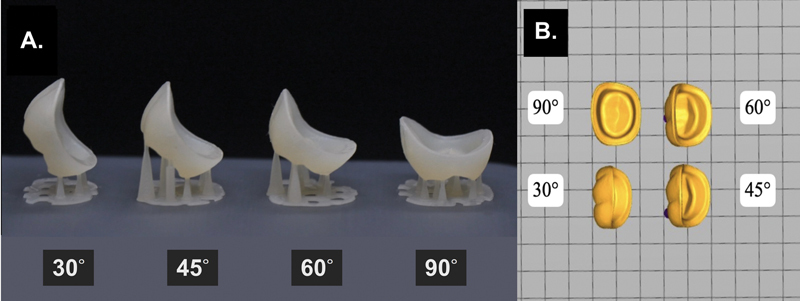
The three-dimensional (3D)-printed crown at the different printed angles groups. ( **A** ) 3D-printed crown at 30-, 45-, 60-, and 90-degree printed angulations between crown's long axis and building plate. ( **B** ) Position of each angulation of 3D-printed crown was placed 5 mm away from the center of the building platform.

The STL file of the die model was used to design the virtual 3D model of the crown restoration using Exocad software (Align Technology, Massachusetts, United States). Parameters were set, including 50 µm cement space and zero marginal gap. To fabricate milled hybrid ceramic crowns, VITA ENAMIC (E; VITA Zahnfabrik, Bad Säckingen, Germany) and Cerasmart (CS; GC Corporation, Tokyo, Japan) were used. The designed restoration STL file was transferred to the milling machine (MC XL, Dentsply Sirona, North Carolina, United States) using fine mode. To produce VarseoSmile Crown ^plus^ (VS; BEGO GmbH & Co., Bremen, Germany) 3D-printed hybrid ceramic crowns, the STL file of the restoration was imported to the DLP 3D printer (ASIGA MAX UV385, Asiga, Sydney, Australia). Printing angles were set at the different long axes of restoration at 30, 45, 60, and 90 degrees to the printing platform, with autogenerated supports as shown in Fig. 2A by using 25 μm layer thickness. Printed crowns were cleaned in 96% ethanol in an ultrasonic bath for 3 minutes, followed by spraying with 96% ethanol as the manufacturer recommended. They underwent postcuring procedure using BEGO Otoflash (BEGO GmbH & Co.) with two cycles of 1,500 flashes each (rotating the object between cycles). To ensure consistency, each printing cycle included all four angulations at the same time, placed 5 mm away from the platform center as shown in Fig. 2B . All fabrication procedures were performed by a single, highly trained operator.

For accuracy and reproducibility assessments were evaluated using the 3D deviation analysis. The crown specimen was all dimensionally scanned with a lab scanner (MEDIT T710, MEDIT). The STL files of scanned crown were imported in the 3D analyzing software (ZEISS INSPECT Optical 3D, GOM Metrology, Braunschweig, Germany). Sprues and supports were virtually removed using the repair mesh command and the resulting STL files of the crown were superimposed with the STL file of previously designed 3D model of the crown through the automatic best-fit alignment mode. The distances between designed 3D model and all scanned crowns for each point were measured using CAD comparison (surface comparison on CAD) command to create color difference map, then 10 corresponding measurement points were randomly selected per surface, including occlusal, mesial, distal, buccal, and lingual, to calculate the root mean square (RMS), following a previous study. 13 Accuracy and reproducibility were analyzed using RMS to quantify deviations. The accuracy refers to the correspondence of the crown with that of the STL file design. The reproducibility refers to the deviation between the first restoration and another. The RMS average values were calculated as follows:



where *x*_ 1, *i*_ are reference data, *x*_ 2, *i*_ are scanned data, and *n* is the number of measuring point.

A color difference map visually represented the findings, with the scale ranging from maximum to minimum deviation. The map demonstrates outward (red) and inward (blue) deviations between superimposed STL files, with minimal displacement (±0.6 µm) represented in green.

**Fig. 3:**
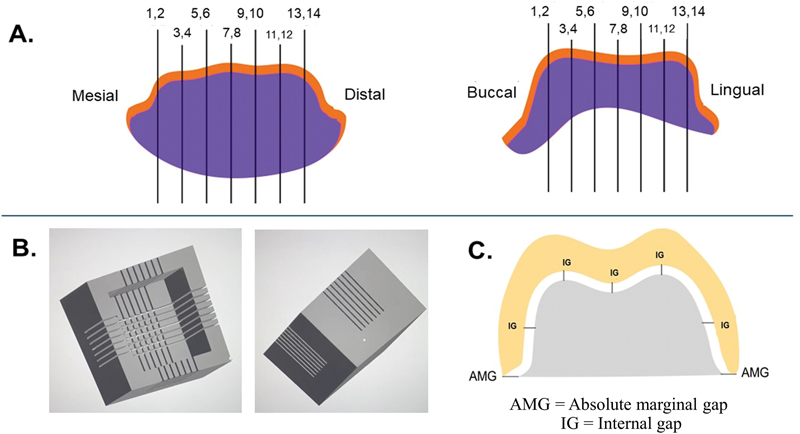
External and internal gap measurement using silicone replica. ( **A** ) Buccolingual and mesiodistal vertical sections of the specimen. ( **B** ) Specially designed rectangular jig with predefined slots. ( **C** ) Absolute marginal gap (AMG) and internal gap (IG) measuring point.

For the assessment of external gap and IG, the silicone replica method was employed. Light body silicone (Aquasil Ultra XLV Regular Set, Dentsply Sirona) was loaded inside the restoration. The restoration was then seated onto the die with a 50 N compressive force using a universal testing machine (Shimadzu AGS-X, Shimadzu, Kyoto, Japan) until the silicone had set. Excess silicone was carefully removed using a No. 12 blade. Afterwards, the restoration, with the intact light body silicone inside, was gently removed from the die. A different color monophase impression material (Aquasil Ultra XLV, Dentsply Sirona) was then loaded into the restoration containing the intact light body silicone. This same monophase impression material was also placed into a rectangular mold. The restoration with the two layers of silicone (light body and monophase) was seated into the rectangular mold filled with the same different color monophase impression material (Aquasil Ultra XLV, Dentsply Sirona). Once this material had set, the silicone replica was carefully removed from the rectangular mold. The silicone replica was repeated twice per specimen for mesiodistal and buccolingual sections. Each replica was then sectioned at the occlusal center parallel to the long axis, and serially at 1 mm intervals bilaterally for a total of eight sections ( Fig. 3A ) using a specially designed rectangular jig with predefined slots to ensure accurate sectioning. ( Fig. 3B ). The jig was constructed to prevent angled cuts and to maintain alignment, ensuring that the sections were parallel to the long axis and minimizing errors during the sectioning of the silicone replicas. The two absolute marginal gaps (AMGs) were measured from the cervical margin of the crown to the preparation margin as the representative of external gap and five IG points measured the perpendicular distance from the internal surface of the restoration to the axial wall of the preparation, of each 14 measurement sides were evaluated ( Fig. 3C ). Measurements were performed under a 20× stereomicroscope (Euromex, Arnhem, the Netherlands) with ImageFocus Plus software (Euromex). Hence, 56 AMG and 140 IG points were evaluated per crown.

The results were analyzed using SPSS 20.0 software for Mac (SPSS Inc, Illinois, United States) at a 95% confidence level and *α* = 0.05. Normality was assumed, but not homogeneity of variances for accuracy, reproducibility, AMG, and IG. Welch's analysis of variance followed by Dunnett's T3 tests were used to compare RMS, AMG, and IG among the groups.

## Results

**Fig. 4:**
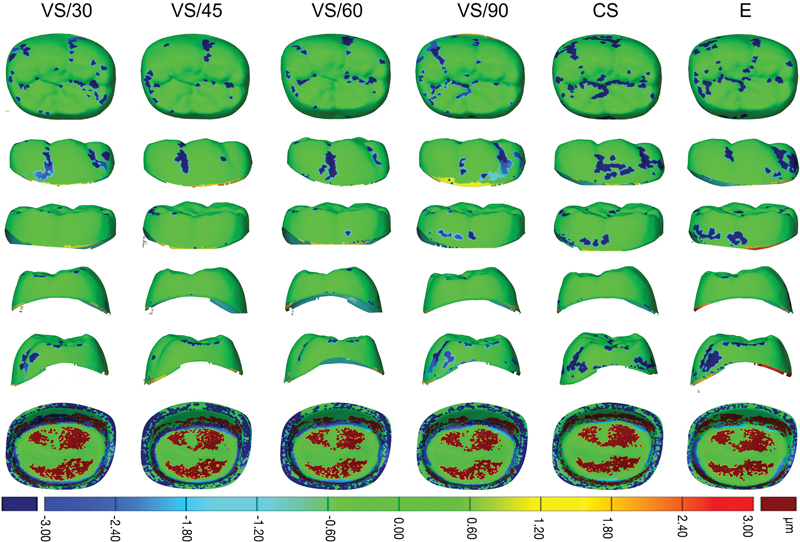
Color difference map of external and internal surface for accuracy analysis for three-dimensional (3D)-printed crown (VarseoSmile Crown ^plus^ [VS]) at 30-, 45-, 60-, and 90-degree printing angulation, and milled crown (Cerasmart [CS] and Enamic [E]).

**Fig. 5:**
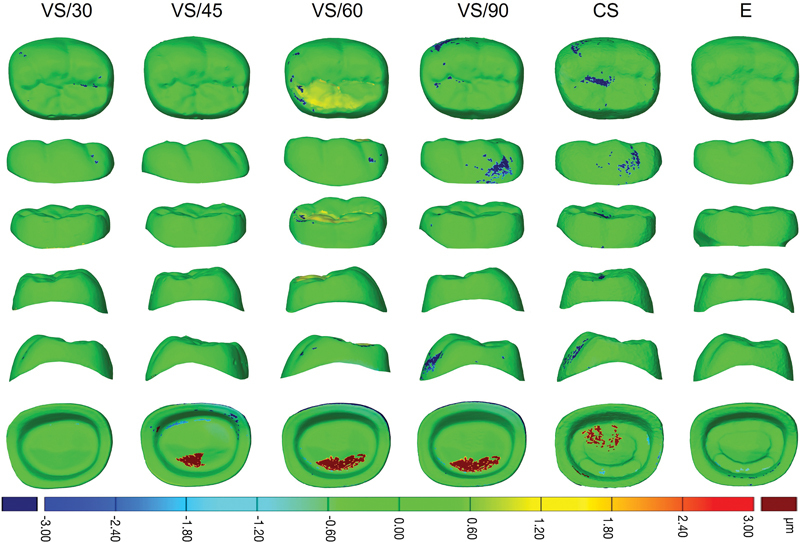
Color difference map of external and internal surface for reproducibility analysis for three-dimensional (3D)-printed crown (VarseoSmile Crown ^plus^ [VS]) at 30-, 45-, 60-, and 90-degree printing angulation, and milled crown (Cerasmart [CS] and Enamic [E]).

The results of the accuracy and reproducibility assessment (RMS) by 3D deviation analysis were summarized in Tables 1 and 2 . For overall external accuracy, no statistically significant differences were found among all groups ( *p* = 0.063). For overall internal accuracy and internal and external reproducibility, significant differences were found among all groups ( *p* < 0.001). The color difference map of accuracy and reproducibility generated for 3D-printed crown (VS) at 30, 45, 60, and 90 degrees, and milled crown (CS and E) are shown in Figs. 4 and 5 , respectively. The results of the marginal gap and IG evaluation by silicone replica technique are summarized in Tables 3 . Statistically significant differences of IG and external gap were found among groups ( *p* < 0.001).

**Table 1:** The accuracy at the external and internal surface of 3D-printed crown (VS) at 30-, 45-, 60-, and 90-degree printing angulation, and milled crown (CS and E)

**Group**	**Mean ± standard deviations (μm) of external accuracy**					
	**Overall**	**Buccal**	**Lingual**	**Mesial**	**Distal**	**Occlusal**
VS/30	0.70 ± 0.13a	2.15 ± 0.20a	0.01 ± 0.00a	0.01 ± 0.00a	0.51 ± 0.04a	0.80 ± 0.06a
VS/45	0.55 ± 0.25a	1.36 ± 0.41a	0.17 ± 0.01b	0.01 ± 0.00a	0.12 ± 0.02b	1.07 ± 0.03b,c
VS/60	0.69 ± 0.38a	1.94 ± 0.62a	0.12 ± 0.01c	0.05 ± 0.01b, c	0.43 ± 0.05a	0.92 ± 0.05a,b
VS/90	1.39 ± 0.82a	3.97 ± 1.04a	0.25 ± 0.02d	0.08 ± 0.01c,d	0.37 ± 0.01a	2.31 ± 0.36c
CS	0.69 ± 0.36a	1.30 ± 0.35a	0.40 ± 0.01e	0.03 ± 0.00b	0.25 ± 0.02c	1.47 ± 0.41a,b,c
E	0.85 ± 0.57a	2.71 ± 0.67a	0.42 ± 0.02e	0.11 ± 0.01d	0.48 ± 0.03a	1.68 ± 0.51a,b,c
*p* -Value	0.063	0.105	< 0.001	< 0.001	< 0.001	0.001
**Group**	**Mean ± standard deviations (μm) of internal accuracy**					
	**Overall**	**Buccal**	**Lingual**	**Mesial**	**Distal**	**Occlusal**
VS/30	14.12 ± 0.97a	17.37 ± 3.67a	19.07 ± 2.41a	15.41 ± 2.96a	15.54 ± 4.06a	3.10 ± 1.77a
VS/45	15.20 ± 1.68a	22.08 ± 6.86a	20.17 ± 3.95a	17.92 ± 3.90a,b	15.63 ± 4.92a	2.58 ± 0.54a
VS/60	14.68 ± 1.16a	18.84 ± 3.28a	19.94 ± 2.59a	17.98 ± 3.32a,b	14.67 ± 5.31a	2.15 ± 1.01a
VS/90	14.09 ± 1.24a	17.94 ± 2.38a	17.95 ± 1.84a	16.51 ± 3.78a,b	16.75 ± 5.10a	1.92 ± 0.31a
CS	20.02 ± 1.97b	18.76 ± 4.21a	33.32 ± 7.02b	19.96 ± 3.12b	27.23 ± 3.10b	0.84 ± 0.58b
E	16.76 ± 2.22a	20.37 ± 5.25a	22.88 ± 6.93a	20.18 ± 4.41b	18.79 ± 4.49a	1.60 ± 0.89a,b
*p* -Value	< 0.001	0.428	< 0.001	0.039	< 0.001	< 0.001

Abbreviations: 3D, three-dimensional; CS, Cerasmart; E, Enamic; VS, VarseoSmile Crown ^plus^ .

Note: Different lowercase letters in the same column indicates significant differences among 3D-printed crowns (VS) at 30-, 45-, 60-, and 90-degree printing angulation, and milled crowns (CS and E) ( *p* < 0.05).

**Table 2:** Reproducibility at the external and internal surface of 3D-printed crown (VS) at 30-, 45-, 60-, and 90-degree printing angulation, and milled crown (CS and E)

**Group**	**Mean ± standard deviations (μm) of external reproducibility**					
	**Overall**	**Buccal**	**Lingual**	**Mesial**	**Distal**	**Occlusal**
VS/30	0.05 ± 0.00a	0.07 ± 0.00a	0.04 ± 0.01a	0.10 ± 0.01a	0.01 ± 0.00a	0.03 ± 0.00a
VS/45	0.10 ± 0.02a,b	0.11 ± 0.02a	0.14 ± 0.04a,b	0.08 ± 0.01a	0.09 ± 0.02a,b,c	0.08 ± 0.02a
VS/60	0.08 ± 0.00b	0.09 ± 0.01a	0.06 ± 0.01a	0.17 ± 0.01b	0.05 ± 0.01a,b	0.04 ± 0.00a
VS/90	0.27 ± 0.01c	0.13 ± 0.02a	0.19 ± 0.04a,b	0.04 ± 0.00c	0.06 ± 0.00b	0.94 ± 0.04b
CS	0.27 ± 0.05b,c	0.37 ± 0.02b	0.13 ± 0.04a,b	0.04 ± 0.00c	0.10 ± 0.03a,b,c	0.74 ± 0.26a,b
E	0.58 ± 0.03d	0.71 ± 0.03c	0.14 ± 0.01b	1.09 ± 0.10d	0.12 ± 0.01c	0.82 ± 0.03b
*p* -Value	< 0.001	< 0.001	< 0.001	< 0.001	< 0.001	< 0.001
**Group**	**Mean ± standard deviations (μm) of internal reproducibility**					
	**Overall**	**Buccal**	**Lingual**	**Mesial**	**Distal**	**Occlusal**
VS/30	0.69 ± 0.04a	0.22 ± 0.02b,c	0.15 ± 0.02b,c	0.22 ± 0.03a	1.42 ± 0.11a	1.46 ± 0.13a
VS/45	0.15 ± 0.01b	0.06 ± 0.01a	0.20 ± 0.03b,c	0.13 ± 0.02a,b	0.03 ± 0.00b	0.32 ± 0.04b,c
VS/60	0.20 ± 0.01c,d	0.21 ± 0.02b,c	0.04 ± 0.01a	0.12 ± 0.02a,b	0.25 ± 0.03c,d	0.39 ± 0.03b
VS/90	0.63 ± 0.09a	0.05 ± 0.01a	0.08 ± 0.01a,b	0.08 ± 0.01b	0.32 ± 0.04c	2.60 ± 0.49a
CS	0.16 ± 0.01b,c	0.26 ± 0.02b	0.11 ± 0.04a,b,c	0.09 ± 0.01b	0.11 ± 0.03b,d	0.22 ± 0.04c
E	0.25 ± 0.02d	0.14 ± 0.01c	0.27 ± 0.04c	0.03 ± 0.01c	0.15 ± 0.03d	0.67 ± 0.07d
*p* -Value	< 0.001	< 0.001	< 0.001	< 0.001	< 0.001	< 0.001

Abbreviations: 3D, three-dimensional; CS, Cerasmart; E, Enamic; VS, VarseoSmile Crown ^plus^ .

Note: Different lowercase letters in the same column indicates significant differences among 3D-printed crowns (VS) at 30-, 45-, 60-, and 90-degree printing angulation, and milled crowns (CS and E) ( *p* < 0.05).

**Table 3:** The external and internal gap of 3D-printed crown (VS) at 30-, 45-, 60-, and 90-degree printing angulation, and milled crown (CS and E)

**Group**	**Mean ± standard deviations of external gap (μm)**					
	**Overall**	**Buccal**	**Lingual**	**Mesial**	**Distal**	
VS/30	93.11 ± 3.13a	133.31 ± 10.51a	78.22 ± 4.66a	91.51 ± 6.08a	69.40 ± 6.46a	
VS/45	84.54 ± 3.68b	105.82 ± 9.05b	76.25 ± 6.65a	82.24 ± 7.59a,b	73.85 ± 5.75a	
VS/60	74.33 ± 3.03c	78.53 ± 5.81c	74.23 ± 5.09a	73.31 ± 4.71b	71.23 ± 7.32a	
VS/90	76.02 ± 3.55c	120.58 ± 9.69a	49.00 ± 5.33b	89.42 ± 5.77a	45.06 ± 5.99b	
CS	45.22 ± 3.19d	44.70 ± 4.66d	40.32 ± 5.09c	50.22 ± 6.68c	45.65 ± 6.03b	
E	44.80 ± 3.57d	43.95 ± 6.35d	50.12 ± 5.44b	41.72 ± 6.19c	42.57 ± 5.31b	
*p* -Value	< 0.001	< 0.001	< 0.001	< 0.001	< 0.001	
**Group**	**Mean ± standard deviations of internal gap (μm)**					
	**Overall**	**Buccal**	**Lingual**	**Mesial**	**Distal**	**Occlusal**
VS/30	38.92 ± 0.63a,b	23.99 ± 2.31a	17.53 ± 3.90a	20.24 ± 2.99a,b	19.34 ± 2.78a	113.52 ± 1.26a
VS/45	37.63 ± 1.07a	21.43 ± 2.96a	20.75 ± 2.42a,b	19.06 ± 2.91a,b	16.81 ± 3.46a,b	110.12 ± 0.18b
VS/60	39.95 ± 0.99b	19.63 ± 3.76a	24.95 ± 2.27c	16.61 ± 2.35a	19.82 ± 2.28a	118.76 ± 0.60c
VS/90	33.76 ± 1.50c	23.66 ± 2.83a	24.43 ± 2.75b,c	20.78 ± 2.654b	11.36 ± 1.94c	88.56 ± 7.26d
CS	53.30 ± 0.95d	45.45 ± 2.28b	7.04 ± 2.02d	39.88 ± 3.09c	24.41 ± 3.10d	149.72 ± 1.71e
E	45.56 ± 0.96e	29.88 ± 2.86c	9.60 ± 2.69d	26.38 ± 1.96d	13.19 ± 2.68b,c	148.75 ± 1.64e
*p* -Value	< 0.001	< 0.001	< 0.001	< 0.001	< 0.001	< 0.001

Abbreviations: 3D, three-dimensional; CS, Cerasmart; E, Enamic; VS, VarseoSmile Crown ^plus^ .

Note: Different lowercase letters in the same column indicates significant differences among 3D-printed crowns (VS) at 30-, 45-, 60-, and 90-degree printing angulation, and milled crowns (CS and E) ( *p* < 0.05).

## Discussion

This study aimed to compare accuracy, reproducibility, and gaps of 3D-printed crowns (VS) at various angles (30, 45, 60, and 90 degrees) with two milled (CS and E) hybrid ceramics. The null hypothesis for external accuracy was accepted but rejected for internal accuracy, reproducibility, and gaps.

The external accuracy across all groups was assessed by mean RMS values, which varied from 0.55 to 1.39 μm. However, the differences observed were not statistically significant ( *p* = 0.063) among the 3D-printed crowns at different angles and milled hybrid ceramic crowns. In the color difference map, higher tolerance levels, such as those reported in previous studies, ± 5 14 and ± 10 μm, 15 16 have been used for analyzing deviations. However, in this study, a more precise tolerance level of ± 0.6 μm was employed for 3D color mapping based on the resolution capabilities of the ZEISS INSPECT Optical 3D system. This value was selected after testing various tolerance thresholds, where result found that ± 0.6 μm provided the most distinct and interpretable visualization of deviations between superimposed STL files. Fig. 4 shows negative deviations, blue color, predominantly appear in the pits and fissures of the buccal and occlusal surfaces, which are attributed to deeper or wider pits and fissures. In contrast, no deviation was detected in the functional contact areas, such as the cusp slopes and fossae, which are represented by green. This suggests that occlusal adjustments may not be required for optimal biting and function. Conversely, the proximal aspects, both mesial and distal, display lower RMS values, which indicate better accuracy as depicted by the predominantly green. This range of proximal RMS values, 0.01 to 0.48 μm, is lower than the periodontal ligament space, suggesting that these measurements likely do not necessitate contact adjustments during the insertion of restorations. Moreover, these proximal measurements do not result in increased contact pressure on adjacent teeth and are not harmful to surrounding tissues. 17 In the gingival third area of the restoration, the buccal, lingual, and proximal sides all exhibited good accuracy, primarily represented by the color green. However, slightly lower accuracy, indicated by positive deviations of less than 4 μm and represented by yellow and red colors, was also observed. According to Ehrlich and Hochman, such deviations in the gingival area whether undercontouring or overcontouring by no more than 1,000 μm (1 mm) do not have clinically significant effects on gingival health. 17

Assessment of the internal accuracy of 3D-printed crowns showed that the mean RMS values were consistently within the range of 14.09 to 15.20 μm across various printing angles, indicating no significant differences. Conversely, milled crowns demonstrated comparatively lower accuracy, with higher RMS values ranging from 16.76 to 20.02 μm, when compared to their 3D-printed counterparts. Both positive and negative internal deviations from the CAD design were observed across all printing angles in 3D-printed crowns and were also found in milled crowns. For 3D printing, the observed deviations can be attributed to material deposition and flow, the nature of the printing process, which involves successive layering, and polymerization shrinkage during printing. 18 Positive deviations, shown as red on the internal surfaces, are pronounced on the curved paths of the cusp slopes on the occlusal surface and the curved regions of the buccal and lingual surfaces, likely due to how the material is deposited. Generally, larger curved surfaces are more prone to the staircase effect than vertical surfaces, leading to greater dimensional inaccuracies. 19 Negative deviation on the internal surface, indicated by blue color, is primarily seen at the gingival margin and the axio-occlusal line angle. In areas with sharp angles, such as the axio-occlusal line angle, negative deviations may occur as material flows away from these regions. Additionally, the light curing process can significantly influence polymerization shrinkage, further impacting dimensional inaccuracies and deviations from the intended CAD design. 18 In milled hybrid ceramic materials, negative deviations at the axio-occlusal line angle are often attributed to machining limitations related to the diameter of the bur used. These errors are predominantly seen when machining angled regions, where the complexities of the internal geometry are more pronounced. 20 Therefore, the positive and negative deviations observed in both the 3D-printed crowns at all different printed angles and the milled crowns did not hinder the clinician during restoration insertion, as they did not exceed the 50-μm cement space established during the CAD phase.

The overall external and internal precision of 3D-printed crowns at different printing angles ranged from 0.05 to 0.27 μm and 0.15 to 0.69 μm, respectively. These findings align with those reported by Nulty, who noted that recent 3D printing devices can achieve accuracies within 30 μm across all dimensions. 21 For the milling group, overall external reproducibility ranged from 0.27 to 0.58 μm, and overall internal reproducibility ranged from 0.16 to 0.25 μm. Bur deterioration during the milling process is a significant factor affecting reproducibility. Although Ceylan et al noted that milling burs used more than four times showed decreased surface integrity when milling zirconia-reinforced lithium silicate, 22 this study achieved excellent reproducibility using the same set of burs to fabricate 10 hybrid ceramic specimens in each milled group. The hardness of the material also influences the lifespan of milling burs, as demonstrated by Al Hamad et al. 23 In addition, reproducibility in hybrid ceramic fabrication has not been widely published. Only one study by Abualsaud and Alalawi, which compared reproducibility or precision between 3D printing and milling techniques on zirconia crowns, reported lower precision than this study, possibly due to material differences. 24 This study found that the best printing angles for both external and internal reproducibility were inconsistent, thus not allowing for a definitive conclusion. In restorative dentistry, reproducibility, defined as the consistency of repeated measurements under unchanged conditions, is generally less crucial clinically due to the rarity of producing identical crowns for the same tooth multiple times. However, reproducibility is vital in processes that require exact replication of measurements, such as in the calibration of CAD/CAM systems. Unfortunately, there is a lack of consensus on a clinically acceptable RMS value for the accuracy and reproducibility of dental restorations. While some publications have used 50 μm 25 26 27 or 100 μm 21 28 as reference tolerances, a standardized value has not been established across the field. In this study, the reproducibility or precision values for both the 3D-printed and milled groups were reported to be less than 3 μm, which is considered clinically acceptable.

This study utilizes the silicone replica method to evaluate both the internal and external gaps between a fabricated crown and a tooth abutment. This approach is adopted because accuracy and reproducibility measurements, which typically compare a restoration's fit to the digital STL design file or among repeated fabrications, may not be fully relevant to clinical scenarios. The silicone replica method involves injecting silicone to precisely measure the internal and external discrepancies, offering a simulation more relevant to clinical settings, especially when cementation techniques are employed to secure the restoration. However, this method can introduce distortions depending on the properties of the impression material. Despite this, Hasanzade et al demonstrated a high level of reliability in assessing the internal and external gaps of restorations, showing comparability between the 3D digital technique and the two-dimensional replica method. 29 An excellent marginal fit is crucial as it reduces the space where bacteria can accumulate, significantly lowering the risk of recurrent caries and periodontal disease. 30 31 Moreover, good internal adaptation is vital for the retention, resistance, and function of the restoration, enhancing its durability and ensuring that it functions well and remains stable over time. 31 Statistical analysis showed that the IGs of the 3D-printed crowns (ranging from 33.76 to 39.95 μm) were significantly lower than those of the milled hybrid ceramic crowns (ranging from 45.56 to 53.30 μm), indicating superior internal fit for the 3D-printed crowns. This finding aligns with results on internal accuracy, which also favored 3D-printed crowns. Furthermore, printing angulation within the 3D printing group influenced IG sizes, with crowns printed at a 90-degree angle showing the smallest internal discrepancies. Conversely, when examining external gaps, milled hybrid ceramic crowns exhibited significantly smaller gaps (ranging from 44.81 to 45.22 μm) compared to 3D-printed crowns (ranging from 74.33 to 93.11 μm). This suggests that the excellent internal accuracy of 3D printing may affect how well the crown fits onto the abutment when tested with silicone, potentially exacerbated by excessive polymerization during the postcuring process of 3D printing. Within the 3D printing group, the angle of printing also influenced the external gaps, with crowns printed at 60- and 90-degree angles exhibiting significantly smaller discrepancies compared to those printed at 30- and 45-degree angles. Despite variations in marginal gap values among the tested groups, influenced by differences in technology (3D printing vs. milling) and printing angulation, the marginal gap measurements across all groups remained within the clinically accepted range, typically considered to be up to 120 µm. 32 33 34 This threshold is associated with the degradation of luting cement, which can lead to microleakage. 35 This outcome is consistent with findings from a previous study by Suksuphan et al, 36 validating the clinical acceptability of the marginal gaps observed in this study. However, this study was conducted under controlled laboratory conditions, limiting its clinical applicability. In this study, fine-mode milling was employed due to its common use in clinical practice, offering a practical balance between speed and accuracy. The results demonstrated that hybrid ceramic crowns milled with fine-mode burs achieved clinically acceptable levels of accuracy, reproducibility, and marginal gap dimensions. However, research has shown that fine-mode milling results in a higher surface roughness compared to the extra-fine mode, which employs smaller-grit diamond burs and may also influence accuracy and gap. 37 Consequently, clinicians may consider utilizing extra-fine burs in cases where enhanced accuracy is required, particularly for achieving optimal fit. It is important to note, however, that the use of the extra-fine mode is associated with longer milling times. Moreover, focusing exclusively on hybrid ceramic materials may restrict generalizability to other dental restoration materials. Factors like flexural strength, fracture toughness, esthetic appearance, color stability, and long-term performance warrant further investigation to fully assess restoration success and durability.
